# Association of a High-Efficiency Particulate Air Filter COVID-19 Testing Booth With Testing Capacity, Cost Per Test Acquired, and Use of Personal Protective Equipment

**DOI:** 10.1001/jamanetworkopen.2021.17698

**Published:** 2021-07-20

**Authors:** Kristian R. Olson, Sandra J. Butler, Bonnie B. Blanchfield, Jean M. Bernhardt, Frank Santo, Nour Al-Sultan, Paul F. Currier

**Affiliations:** 1 Department of Medicine, Massachusetts General Hospital, Boston; 2 Department of Pediatrics, Massachusetts General Hospital, Boston; 3 Harvard Medical School, Boston, Massachusetts; 4 MGH Springboard Studio, Boston, Massachusetts; 5 Department of Health Policy and Management, Harvard T.H. Chan School of Public Health, Boston, Massachusetts; 6 Department of Medicine, Brigham and Women’s Hospital, Boston, Massachusetts; 7 Division of General Internal Medicine, Massachusetts General Hospital, Boston; 8 Division of Pulmonary and Critical Care Medicine, Massachusetts General Hospital, Boston

## Abstract

This quality improvement study compares COVID-19 testing throughput, personal protective equipment use, and cost per test before vs after the introduction of high-efficiency particulate air–filtered, positive-pressure Hexapod personal protection booths at a hospital in Boston, Massachusetts.

## Introduction

**Figure 1.**
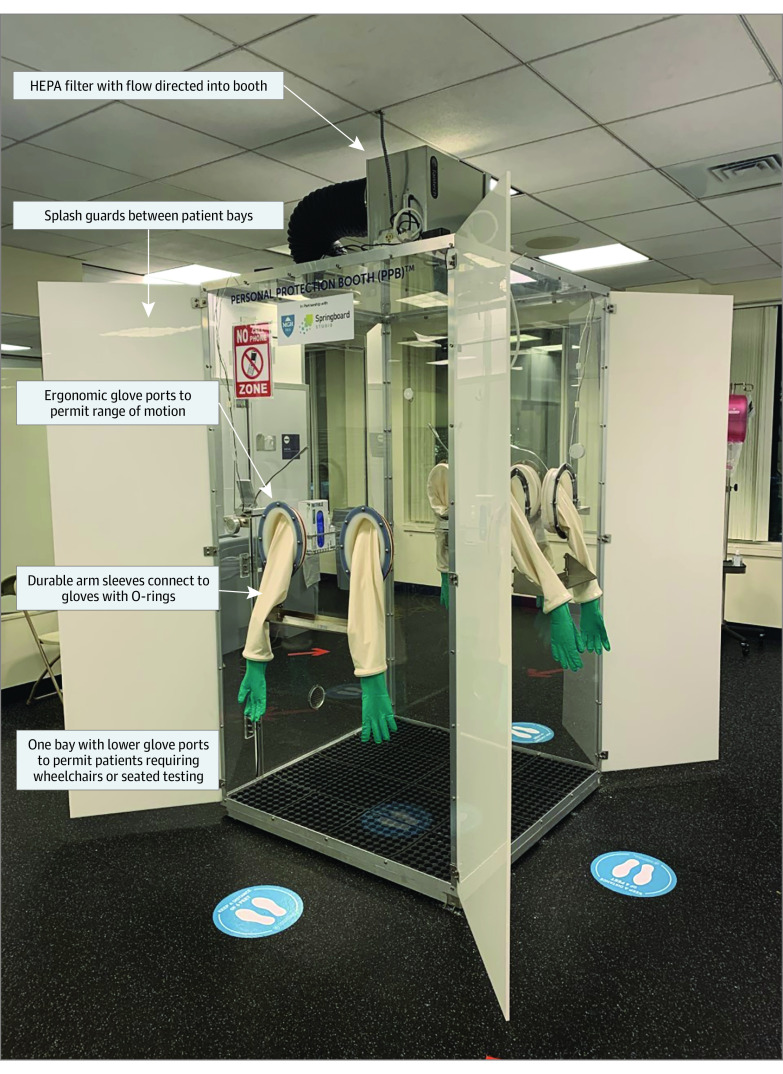
Hexapod Personal Protection Booth

In March 2020, COVID-19 testing demand and scarce availability of personal protective equipment (PPE) were concerns for Mass General Brigham (MGB) in Boston. In response, an in-hospital innovation unit engaged frontline clinicians, designers, and engineers to design a solution for outpatient testing to improve throughput and conserve PPE. The result was a high-efficiency particulate air–filtered, positive-pressure personal protection booth with 6 ergonomic glove ports called a Hexapod. Efficiency was gained by using 3 patient bays, 1 of which was wheelchair accessible, for each individual obtaining swab samples. The final model was in use within 28 days (Figure 1).^1,2^ Training for new users typically required less than 30 minutes. We assessed the utility of Hexapods for COVID-19 testing.^3^

## Methods

A design-thinking approach incorporating observation of nurses, sanitation personnel, and infection control workers at MGB was used for the development of the Hexapods. Learnings were incorporated iteratively until a final model was deployed. Beginning April 16, 2020, 3 final Hexapods were sequentially implemented at MGB. This retrospective quality improvement study, conducted from March 11, 2020, to October 1, 2020, compared COVID-19 testing throughput, PPE use, and cost per test before vs after the introduction of the Hexapods. Staff, PPE use, and daily tests were observed by study staff before and after use of the Hexapods. The cost per day of staff, glove, gown, and consumable supplies used in the Hexapods was calculated and scaled by the number of daily tests. Cost data were obtained from MGB. Analyses were performed using Microsoft Excel, version 16.49. This innovation project met criteria as a quality improvement initiative by the Mass General Brigham Human Research Committee. Informed consent was not required because there were no human experience interviews and data were already routinely collected at the hospital. The study followed the Standards for Quality Improvement Reporting Excellence (SQUIRE) reporting guideline.

## Results

Before implementation of the Hexapods, the maximum testing capacity per testing team in a 9-hour period was 54, with 1 patient scheduled every 10 minutes. After implementation of the Hexapods, testing capacity per day scaled, with a mean (SD) daily throughput of 93 (57.6) tests per booth (72% increase) during 311 booth-days and 28 948 tests performed during the evaluation period. The maximum number of tests during a 9-hour day was 245 (354% increase), reflecting a potentially steady amount of testing as demand increased over time.

Testing for each Hexapod required 5 staff members compared with 4 for each testing team before Hexapod implementation. Despite higher total personnel costs, the increase in testing capacity was associated with a decrease in the personnel cost per test from $28.78 before Hexapod implementation to $18.18 at 93 tests per day and $6.90 at 245 tests per day after implementation.

**Figure 2.**
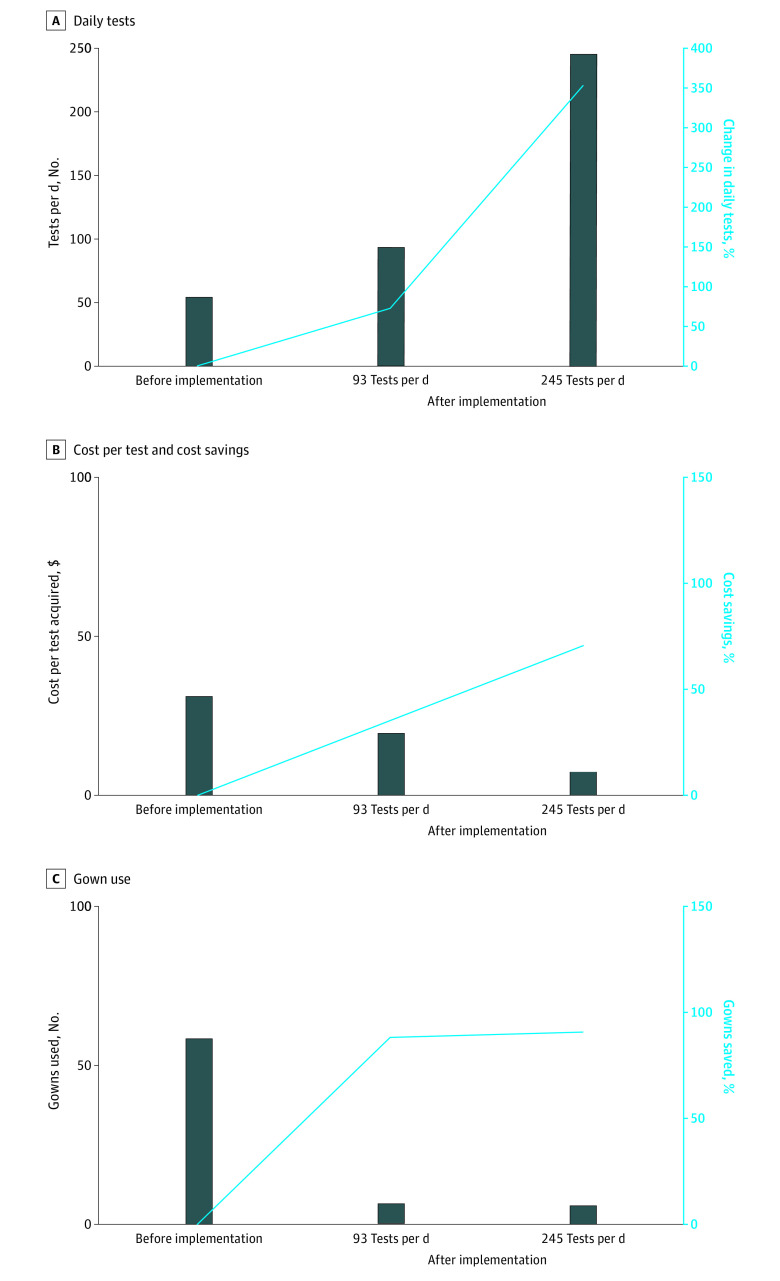
Throughput, Cost Savings, and Gown Use Before vs After Implementation of Hexapod Personal Protection Booths Before implementation of the Hexapods, the maximum testing capacity per testing team in a 9-hour period was 54, with 1 patient scheduled every 10 minutes. After implementation of the Hexapods, the mean (SD) daily throughput was 93 (57.6) tests per booth during 311 booth-days and 28 948 tests performed during the evaluation period. The maximum number of tests during a 9-hour day was 245.

Use of Hexapods was associated with a decrease in disposable gown use of 93.5% at 93 tests per day and 97.6% at 245 tests per day (Figure 2) because the person who obtained the swab sample did not need to change gowns between patients. However, more gloves were used after implementation of the Hexapods because 2 additional personnel, a processor and a cleaner, required glove changes after each patient. Monthly consumable supplies for each Hexapod included 12 pairs of durable gloves and 3 pairs of arm sleeves with O-ring attachments; a new high-efficiency particulate air filter was needed every 3 months. The net PPE costs, including consumable expenses, were lower after implementation of the Hexapods than before implementation ($1.98 before implementation vs $0.62 at 93 tests per day and $0.36 at the 245 tests per day after implementation).

Despite the additional staffing, glove use, consumables, and capital costs required with use of the Hexapods, the increased throughput of the Hexapods was associated with decreased cost per test ($30.77 before implementation vs $19.28 at 93 tests per day and $7.44 at 245 tests per day after implementation). The capital cost of a Hexapod was recouped in 13 days at 93 tests per day and 2 days at 245 tests per day. The annual return on investment was 22.4 at 93 tests per day and 124.3 at 245 tests per day.

## Discussion

In this quality improvement study, Hexapod use was associated with cost savings, decreased use of PPE, and increased testing throughput, suggesting that the use of the Hexapods helped address the aforementioned testing challenges. Engaging frontline staff in human-centered design was associated with a rapid and adoptable solution.^4,5^ A limitation of this study is that sites with different demand and cost structures may realize different cost savings. Because the Hexapods were implemented from concept through evaluation in 6 months, this study suggests that design-thinking approaches may be useful for rapid health care innovation during a crisis and for every day challenges.
